# Sessile Drop Method: Critical Analysis and Optimization for Measuring the Contact Angle of an Ion-Exchange Membrane Surface

**DOI:** 10.3390/membranes12080765

**Published:** 2022-08-04

**Authors:** Maria Ponomar, Ekaterina Krasnyuk, Dmitrii Butylskii, Victor Nikonenko, Yaoming Wang, Chenxiao Jiang, Tongwen Xu, Natalia Pismenskaya

**Affiliations:** 1 Membrane Institute, Kuban State University, 149 Stavropolskaya St., 350040 Krasnodar, Russia; 2 Department of Applied Chemistry, Anhui Provincial Engineering Laboratory of Functional Membrane Science and Technology, School of Chemistry and Materials Science, University of Science and Technology of China, Hefei 230026, China

**Keywords:** surface characterization, ion-exchange membrane, contact angle, sessile drop method, roughness

## Abstract

The contact angle between a membrane surface and a waterdrop lying on its surface provides important information about the hydrophilicity/hydrophobicity of the membrane. This method is well-developed for solid non-swelling materials. However, ion-exchange membranes (IEMs) are gel-like solids that swell in liquids. When an IEM is exposed to air, its degree of swelling changes rapidly, making it difficult to measure the contact angle. In this paper, we examine the known experience of measuring contact angles and suggest a simple equipment that allows the membrane to remain swollen during measurements. An optimized protocol makes it possible to obtain reliable and reproducible results. Measuring parameters such as drop size, water dosing speed and others are optimized. Contact angle measurements are shown for a large number of commercial membranes. These data are supplemented with values from other surface characteristics from optical and profilometric measurements.

## 1. Introduction

Ion exchange membranes (IEMs) are increasingly used in modern technology, applied in chemistry, energy, medicine and other fields [1,2,3]. They constitute the main part of membrane bioreactors [4], fuel cells [5,6] (including biological ones [7]) and other energy generating devices [8], form membrane stacks in dialysis [9] and electrodialysis [10] units, and are used in membrane capacitive deionization [11,12], microfluidics [13,14], potentiometric sensors [15] and others [16]. The degree of hydrophilicity/hydrophobicity of the IEM surface is an important characteristic that affects their performance in the above processes; the membrane permselectivity [17], the ability to increase mass transfer rate due to electroconvection [18,19], fouling resistance [20,21,22] strongly correlate with the degree of surface hydrophilicity. Changes in this characteristic, estimated from contact angle measurements, can be used as an indicator of changes in the chemical composition and structure of the surface due to its modification [23] or membrane degradation during use [24]. These changes can be also caused by the adsorption of organic and inorganic substances by the membrane surface [25,26,27,28], changes in the surface charge [29,30] and/or roughness [20,29].

**Figure 1:**
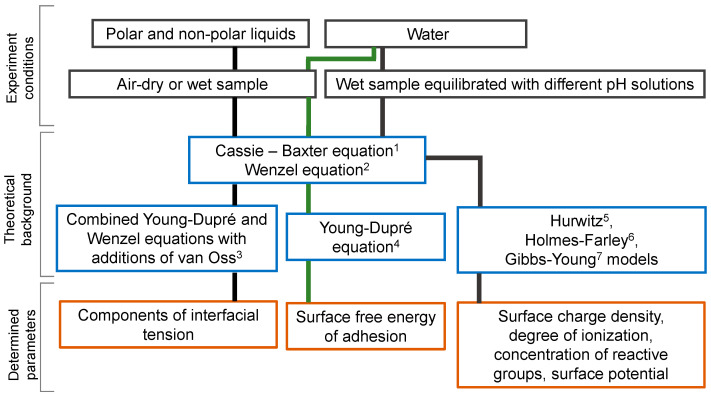
Schematic representation of the possibilities of the contact angle measurement method for determining various parameters of the IEM surface: 1—[38]; 2—[32]; 3—[20,35], 4—[34]; 5—[30]; 6—[36]; 7—[37].

The high significance of the contact angle explains the high popularity of this research method [31]. A Scopus “contact angle” keyword search reveals about 130,000 publications, including about 2400 publications related to IEM (accessed 15 June 2022). Figure 1 shows some possibilities of determining the ion-exchange membrane surface parameters by the contact angle measurement. Thus, the parameters of geometric and chemical heterogeneity combined with the contact angle of the surface under study allow calculating the equilibrium contact angle using the Wenzel and/or Cassi–Baxter equations [32,33]. From this value, the free energy of adhesion (free energy of hydration) can be calculated by the Young–Dupré equation [34]. This energy determines the degree of the surface hydrophilicity/hydrophobicity, which is useful for the evaluation of different methods of membrane modification. If test fluids have different surface tension and chemical nature, information about interfacial tension and its components can be obtained [29,35]. These data are important in the research of fouling and scaling of IEM surfaces [26,27]. Contact angle titration in accordance with [30,36,37] makes it possible to calculate the surface charge density, degree of surface ionization, concentration of reactive groups on the surface and other parameters.

Many approaches have been developed to measure the contact angle [39]. The most common is the sessile drop method. It consists of applying a drop of distilled water (or other test liquid) over the sample surface and measuring the contact angle formed at the three-phase interface (solid–liquid–gas). The sessile drop method is widespread due to the simplicity of the measuring setup and measurement protocol, small volume of the test liquid and size of the sample required for measurements. The method makes it easy to obtain reproducible results for solid non-swelling, geometrically and chemically homogeneous materials (such as polyethylene and polytetrafluoroethylene) [40]. 

The correct contact angle determination is an intensively discussed topic in the literature. Refs. [41,42,43] provide the protocols for measuring the contact angles, which allow obtaining reliable and reproducible data for solid non-swelling surfaces. Our work is aimed at optimizing the contact angle measurement for IEM surfaces, which have special properties. IEMs are semi-solid gel-like polymers that swell in an aqueous medium. Without contact with water, the swelling degree decreases rapidly with time, making it very difficult to determine the correct contact angle value. The values reported in the literature for the same membrane can vary considerably from one publication to another. For example, the contact angles of the Neosepta AMX membrane (manufacture Astom, Japan), reported by different authors in the literature, differ by 20 degrees: 64 [44], 66–70 [45], 69 [46], 80.6 [47] and 84 [48]. The reason for these variations is that researchers use dry and swollen samples for measurements (sometimes without specifying the state of the sample under study). A feature of IEM is that its structure, including the surface layer, strongly depends on the swelling degree [49]. In the air-dry state, the surface of these membranes tends to absorb a drop of polar test liquid (e.g., water) due to the ion–dipole interaction of liquid molecules with fixed IEM groups [49,50]. With that, the surface of the initially dry IEM is deformed in most cases. The value of the contact angle can also be influenced by the composition of the equilibrium solution [51], surface roughness [32], distribution of conductive and nonconductive regions [33,38], drop size [52], the distance between the needle and sample [53], dosing flow rate [41], image registration and processing method, contact angle acquisition [54], time after drop deposition at which the measurement is made [53], and some other details of the measurement procedure [41,43,53,55].

This paper examines the known experience of measuring the contact angles of ion-exchange membranes and proposes an optimized protocol of the sessile drop method. The measurement setup is improved, so that the membrane remains wet during the time of measurements. This makes it possible to obtain reproducible results under conditions when the membrane surface is not deformed. Measurement conditions, such as drop size and dosing flow rate, are optimized. The results of contact angle measurements for a large number of commercial membranes are presented. These data are supplemented by surface characterization using optical microscopy and profilometry. We believe that the optimized protocol can serve as the basis for the standard for measuring contact angles.

## 2. Theoretical Foundations

The sessile drop method is used to obtain an apparent contact angle $\theta_{ap}$, which describes metastable state of a liquid drop on the surface of a real sample. In general, $\theta_{ap}$ is a random value, since the metastable state of two successive droplets may be different [41,52]. The values of $\theta_{ap}$ belong to the range of contact angle hysteresis, which is limited by advancing *θ_A_* and receding *θ_R_* contact angles [56].

**Figure 2:**
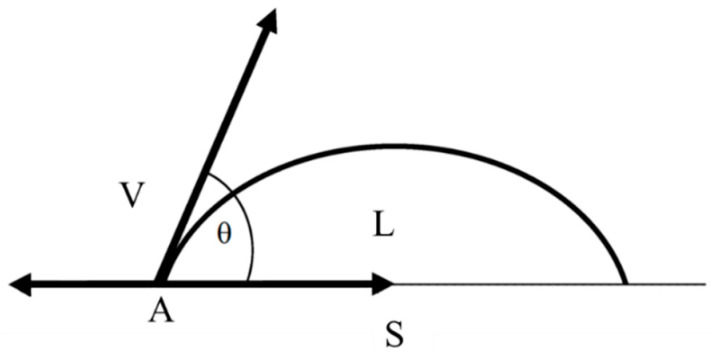
A sessile drop on the solid surface: L, V and S denote liquid, vapor and solid phases, respectively; θ is the contact angle.

The basic relationship linking the wetting properties of the liquid with the contact angle is the Young equation [57]. It expresses the balance of surface forces at point A of contact of the three phases: liquid, solid and gas (Figure 2):

(1) $$\begin{array}{c} \cos\theta^{Y}=\frac{\gamma_{SV}-\gamma_{SL}}{\gamma_{LV}}\; \end{array}$$

where $\theta^{Y}$ is the contact angle of the liquid–solid–gas system; and $\gamma_{SV}$, $\gamma_{SL}$ and $\gamma_{LV}$ are the interfacial tensions in the solid/gas, solid/liquid and liquid/gas systems, respectively. 

The Young equation is valid for an equilibrium system with a perfectly smooth, chemically homogeneous, nonreactive and nondeformable surface [32,33,51]. However, real samples have geometrical and chemical defects. The Wenzel equation was proposed for rough surfaces; it takes into account the change in surface energy caused by the growth of the phase contact area [32,58]:

(2) $$\begin{array}{c} \cos\theta_{ap}=r\cos\theta^{Y} \end{array}$$

Equation (2) relates Young’s contact angle and the apparent contact angle to the roughness factor *r*; *r* is the ratio of the real surface area, A_rough_, to its flat projected area, A_flat_; *r* = A_rough_/ A_flat_. It follows from Equation (2) that, for surfaces with roughness factor *r* > 1, the value of $\theta_{ap}$ > $\theta^{Y}$, if the material is hydrophobic ($\theta^{Y}$ > 90 degree) and $\theta_{ap}$ < $\theta^{Y}$, if the material is hydrophilic ($\theta^{Y}$ < 90 degree).

To account for the change in surface energy resulting from the contact of the test liquid with the areas of a flat surface having different chemical nature, Cassie and Baxter proposed an equation containing the energy contribution of each specific surface area to the formation of the contact angle [33]:

(3) $$\begin{array}{c} \cos\theta^{C}=f_{1}\cos\theta_{1}^{Y}+f_{2}\cos\theta_{2}^{Y} \end{array}$$

where $f_{1}$ и $f_{2}$ are the fractions of the surface areas characterized by Young’s contact angles $\theta_{1}^{Y}$ и $\theta_{2}^{Y}$, respectively.

The equations presented above are widely used to interpret the contact angles. Other equations (also mentioned in Figure 1) have important applications for a more detailed characterization of surface properties. The derivation and analysis of these equations can be found in reviews [31,59].

## 3. Experiment

### 3.1. Membranes

Commercial homogeneous Neosepta CMX, CSE, AMX and ACM membranes (manufacturer Astom, Tokyo, Japan); homogeneous CJMC-2, CJMC-3, CJMC-4, CJMA-2, CJMA-3 and CJMA-4 membranes (manufacturer Hefei Chemjoy Polymer Material, Hefei, China); and heterogeneous MK-40, MA-41 membranes (manufacturer Shchekinoazot, Pervomayskiy, Russia) and Ralex CMH PES, Ralex AMH PES membranes (manufacturer MEGA, Drahobejlova, Czech Republic) were studied. Some of the characteristics of the membranes are presented in Table 1.

The membranes underwent a standard pretreatment procedure [51]. Prior to surface characterization, the samples were equilibrated in 0.02 М NaCl.

### 3.2. Measurement of Membrane Surface Roughness

**Figure 3:**
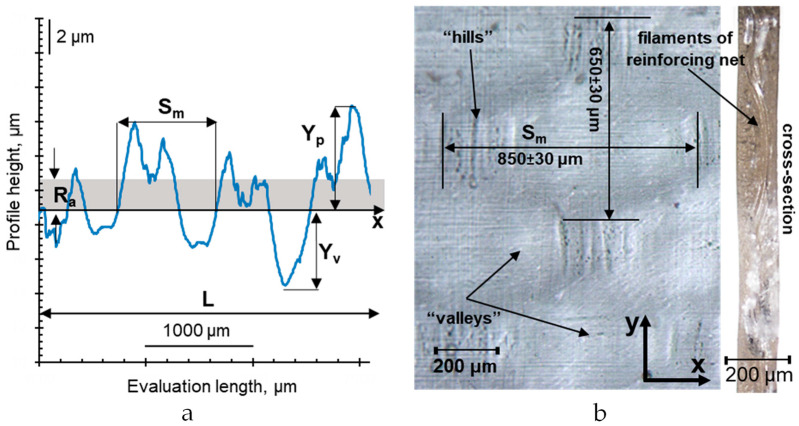
Surface profilogram of the swollen AMX membrane scanned along the x direction in the optical surface image (**a**); optical surface image and cross section of the swollen AMX membrane (**b**).

The roughness profile of the swollen membranes under study was characterized by the following parameters (Figure 3a) [68]:

1. *R_a_* (arithmetic mean roughness) is the arithmetical mean deviation of the assessed profile within the evaluation length:

(4) $$\begin{array}{c} R_{a}=\frac{1}{n}\sum_{i-1}^{n}\left|y_{i}\right| \end{array}$$

where *y_i_* is the ordinate of the *i* point belonging to the profilogram within the evaluation length.

2. *R_t_* is the height distance between the deepest valley, *Y_v_*, and the highest hill, *Y_p_*, within the evaluation length (total height of profile):

(5) $$\begin{array}{c} R_{t}=Y_{p}{}_{max}+Y_{v}{}_{max} \end{array}$$

3. *S_m_* is the mean width of the profile elements (the average distance between the points of intersection of hills with the middle line within the evaluation length):

(6) $$\begin{array}{c} S_{m}=\frac{1}{n}\sum_{i-1}^{n}x_{S_{i}} \end{array}$$

4. *r* is the roughness factor is the ratio of the length of the real surface profile to the length of its geometrical projection. In this work, it was determined as the ratio of the average (found by 10 measurements) real roughness profile length, *L_r_*, to its projection, *L*:

(7) $$\begin{array}{c} r=\frac{L_{r}}{L} \end{array}$$

The value of *L_r_* was found as the sum of distances *L_i_* between each two points on the profilogram by the formula:

(8) $$\begin{array}{c} L_{r}=\sum L_{i}=\sum \sqrt{{\left(x_{i+1}-x_{i}\right)}^{2}+{\left(y_{i+1}-y_{i}\right)}^{2}} \end{array}$$

The above-mentioned surface roughness parameters of the swollen IEM were determined using a TR200 portable roughness meter. The measurements were made in 5000 μm sections in two mutually perpendicular directions: *x* and *y* along the surface of the IEM (Figure 3b). The obtained results within both directions were averaged to obtain the average size of the geometric heterogeneities of the IEM surface. The results of measurements along *x* and *y* may differ due to features of the manufacturing, sample preparation, swelling, etc. To avoid drying and deformation of the membranes, the sample under study was placed on a drop of an equilibrium solution. The number of measurements was equal to at least 3 on different surface areas of each sample. The accuracy of measurements was ±1%. The size of the studied area was commensurate with the diameter of the drop applied to the surface, so the information obtained characterizes the scale of heterogeneities on the surface, which can affect the contact angle.

The use of this method makes it possible to overcome some limitations of such high-resolution methods of surface morphology research as atomic force microscopy (AFM) and scanning electron microscopy (SEM), which are widely used to study IEM [60,69]. The scan size in AFM method, as a rule, does not exceed 100 × 100 microns [70]. In the case of heterogeneous and/or inert material-reinforced membranes, this area is often smaller than the geometric heterogeneity of the samples, and the results depend on the size of the scan [20]. SEM is most often used to study dry samples, whose the surface topography differs significantly from that of swollen membranes [71]. Low-vacuum SEM [72], which can be used without pre-drying the sample, yields large errors due to rapid changes in the surface parameters of swollen ionomers in contact with air. Optical microscopy makes it possible to measure the roughness parameters of IEM cross-sections of any length [69]. However, cutting samples leads to the disruption of membrane structure integrity (first of all, reinforcing fabric) and distortion of surface roughness parameters as compared to those existing in reality.

### 3.3. Visualization of Membrane Surface and Cross Sections

An optical microscope SOPTOP CX40M (Yuyao, Zhejiang, P.R. China) with a digital USB camera was used to visualize the surface and cross section of swollen ion-exchange membranes. The sample was placed on a drop of distilled water situated on the slide table. This prevented the sample from drying out during image acquisition.

### 3.4. Sessile Drop Method of Contact Angle Measurement

#### 3.4.1. Optimized Protocol for Contact Angle Measurement

When using the sessile drop method, the measured contact angle can be influenced by some groups of factors presented in Table 2 [41,43,53,59,73,74]. The optimization of the experimental conditions and the components of the setup for determining the contact angles of IEMs with regard to these factors is presented in Section 3.4.2 and Section 3.4.3. The found optimal parameters for determining the contact angles of the surface of swollen ion-exchange membranes are presented in Table 2.

**Figure 4:**
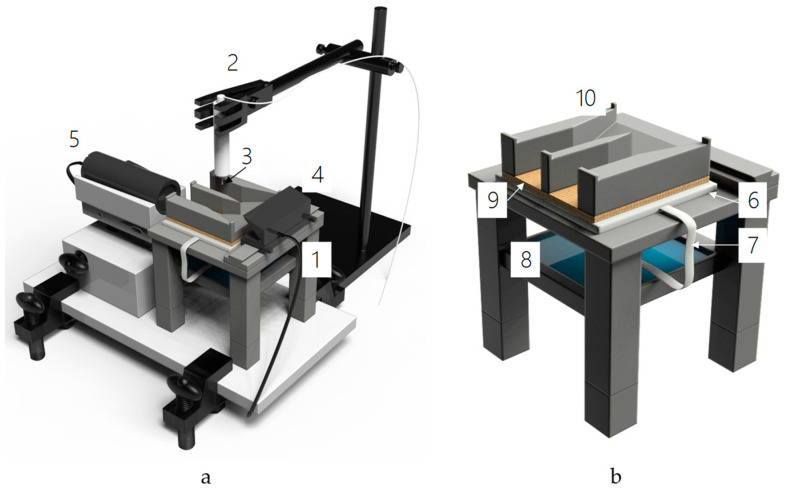
Installation for measurement of contact angles (**a**) and enlarged image of the slide table with a device for keeping the membrane sample wet during measurements (**b**). The setup includes: a slide table (1), a system of dosing the test liquid (2) with a dosing needle (3); a device for providing background lighting of the sample surface (4); a digital microscope DinoLite Pro (2–60× magnification, 1.5 MP) (5). The slide table is equipped with a moist porous substrate (6) connected with a filter strip (7) to a reservoir with distilled water (8) and a pressure plate (10) for keeping the IEM sample (9) moist. The unit is placed on a solid base, whose the position is strictly horizontal.

Figure 4 shows an experimental setup assembled taking into account the advantages and disadvantages of commercial installations used to measure contact angles, including Contact Angle Goniometer (Ossila Ltd., Sheffield, UK) [75], Optical Tensiometer Theta Lite (Biolin Scientific AB, Västra Frölunda, Sweden) [76], OCA 15 IEC (DataPhysics Instruments GmbH, Filderstadt, Germany) [77] and Drop Shape Analyzer DSA100S (KRÜSS GmbH, Hamburg, Germany) [78].

In accordance with the optimized conditions (Table 2), the measurements of the contact angle of the swollen IEM were performed as follows. The moist substrate (6) on the slide table (1) was connected to a reservoir (8) containing distilled water [79], to maintain the moist state of the sample. The dosing system allows varying the speed of applying a drop of the test liquid (distilled water, 2 μS/cm; pH 4.8 in the studied cases); the volume and the height from which the drop falls on the test sample can also be varied. The dosing needle (3) was placed at a distance of 4 mm from the surface of the test sample, and the speed of the syringe pump was set to 0.055 mL/min. The digital microscope (5) was tilted 1.5° to the horizontal surface of the slide table. Pre-equilibrated with the working solution, the swollen sample of IEM (9) was placed on a substrate and fixed with a pressure plate (10). A filter paper was used to remove excess liquid from the surface of the sample to prevent dripping due to excess moisture. The pump was started, a drop of distilled water was applied to the surface of the test sample, and video was recorded using a digital microscope. The frames extracted from the video at specified time intervals after the drop application were processed using the Rising View software. All measurements were performed at 25 ± 0.2 °C, with at least 20 measurements at different sites of the sample. The mean apparent value of the contact angle *θ_ap_* was determined using the t-criterion, and data outliers were controlled using the Grubbs criterion.

#### 3.4.2. Minimizing Errors in Determining Contact Angles

**Method of dosing drop**. The technique of dosing drop determines the speed of drop formation, reproducibility of its volume and shape. As a rule, syringes with removable metal needles or mechanical liquid dispensers with plastic tips are used in installations for measuring contact angles; manual dosing or automated syringe pumps are applied [43,75,76,77,78]. Syringe pumps are highly accurate and avoid operator input into the droplet formation mode, but require the prior selection of optimal operating parameters. In this experiment, a DIXION syringe pump with a 50 mL syringe, which was connected by a tube to a metal needle fixed in the foot of a tripod, was used.

**Figure 5:**
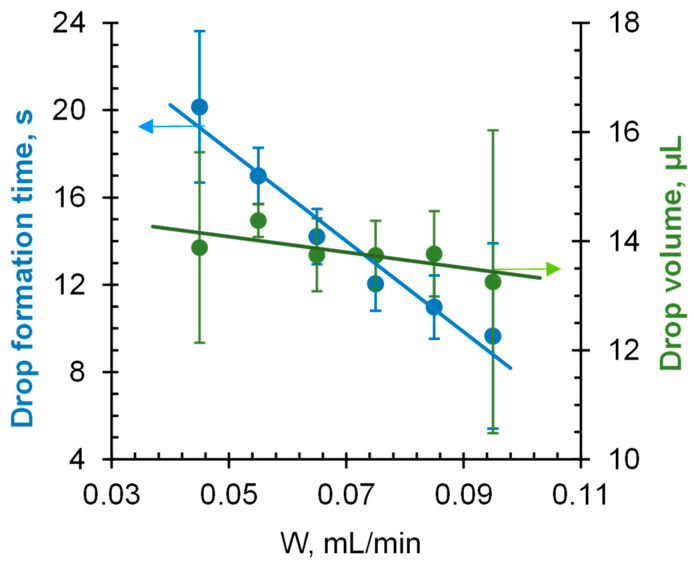
Dependencies of drop volume and drop formation time on volume rate of distilled water delivery by a syringe pump (W) using a 20G dosing needle. The vertical lines on the markers correspond to the confidence interval of the measurement.

**Drop volume.** It is known [41,43] that the droplet volume should not be too small, since small droplets are susceptible to vibration, evaporation, and optical errors. In addition, the larger the ratio of the droplet base size to the average size of geometric or chemical inhomogeneities of the IEM surface, the more reproducible results can be obtained [52,53]. At the same time, the larger the droplet, the more gravity distorts its shape [41]. The typical droplet volume range for the sessile drop method is 3 to 20 µL [41]. To ensure such volumes and the formation of axisymmetric droplets [53], we used a dosing metal needle cut perpendicular to its central axis (pst3 tip type). In accordance with the recommendations presented in [43], the tip of the needle was rubbed with paraffin before the measurements so that the material from which the needle was made would not affect the results. The time of drop formation must be constant during the measurements, since this parameter directly affects the volume of the drop, which is expected to be the same for each drop. Accordingly, the confidence interval when determining this time experimentally must be minimal. Several needles with inner diameters of 280 to 1000 μm and outer diameters of 400 to 1300 μm were tested. The lowest relative error in drop volume and drop formation time measurements was obtained for the needle with the inner and outer diameters of 600 and 800 μm (size 20G), respectively. The drop volume and drop formation time as functions of the drop dosing rate for this needle are shown in Figure 5.

The relative error of the values increases at the extreme limits of the range of W values under study (Figure 5). At small W values, the drop formation time and volume can vary significantly due to the increased effect of random factors (random vibrations of the setup, air currents) on a large unstable drop attached to the needle. At great W values, it is the effect of random changes in the rate of water supply to the needle, which is increased; in addition, the shape of the drop is distorted under the action of gravity. A minimum relative error of <0.5% for drop volume (and <1.5% for drop formation time) is achieved at a volume rate of 0.055 mL/min, which corresponds to a drop volume of 14.4 µL.

**Image registration**. The digital microscope was tilted 1.5° to the horizontal surface of the slide table to obtain a clarity image of the drop profile. This inclination corresponds to those used in commercial installations and does not lead to significant errors when measuring the contact angle [74]. The location of the droplet in the center of the view captured by the microscope provides a more accurate determination of the baseline and interfaces of the liquid and solid phases with the gas phase.

**Background light** was provided by an LED source with a diffuser, which was placed on the slide table behind the sample and directed at the drop. The lighting was chosen so as to minimize the blurring of the three-phase contact boundaries and optical distortions.

**Figure 6:**
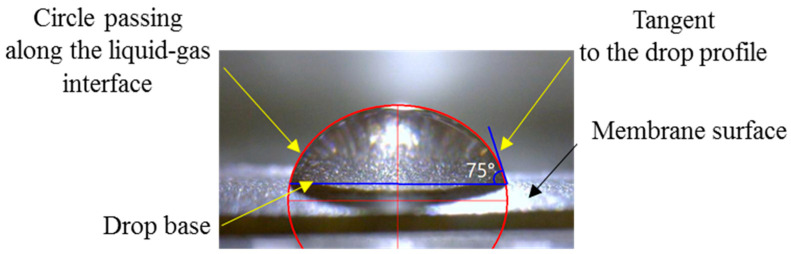
Example of determining *θ_ap_* for an air-dry MK-40 membrane.

**The methods of image processing** have been the subject of many years of scientific development. These methods have a great influence on the result of determining the contact angle, with the accuracy depending on the image resolution and edge detection method. There are many approaches for determining the contact angle in an image based on the droplet profile. The most commonly used methods in commercial software are droplet profile fitting using the Young–Laplace equation [80], circle and ellipsoidal droplet model [81] or polynomial functions [82]. An assessment of the existing image processing methods for digital evaluation of the contact angle is presented in Ref. [54]. One of the common semi-automatic methods of *θ_ap_* determination is the fitting method, where the droplet profile is fitted to a geometric figure (ellipse, circle) (Figure 6), the borders of which run along the “liquid–gas” phase section. Then, *θ_ap_* is measured by aligning a tangent line to the drop profile at the three-phase contact point. In this paper, RisingView is used as image acquisition software. This method does not require any additional commercial software; it is very simple and rather reproducible (∆ < 5%), despite the semi-automatic regime. The results of the contact angle determination by this method can be verified according to the error detection guidelines [53,74].

Distance between the needle and the sample. It is known [53] that obtaining a stable droplet shape requires minimizing the kinetic energy input to the droplet during the dispensing process. This is possible when the dosing needle is positioned at a minimum distance from the sample under study; however, the latter should exceed the droplet diameter: the hanging drop must not touch the surface of the sample [41]. The maximum diameter of the drop hanging at the needle tip in all our measurements was ≈ 3.5 mm. To determine how the distance between the tip of the needle and the sample affects the contact angle, we measured this characteristic for some materials often used as references [74] at various distances between the sample and the needle. The results are shown in Table 3. In accordance with the recommendations presented in [43], the surface of the standard samples was polished and cleaned of adsorbed substances before the measurements to prevent distortions caused by surface roughness or associated with the generation of static electricity. Between measurements, the surface of the samples was gently wiped with a soft hydrophilic cloth, and the remained droplets were blown off with an air stream.

It follows from the data presented in Table 3 that the measured values of *θ_ap_* are close to the literature values. When the distance from the studied sample to the dosing needle was increased in the range from 4 to 6 mm, the difference between the measured and reference contact angles was no more than 3%. However, the error in determining the contact angle increases as the height of drop fall increases. As expected, the maximum reproducibility of results was provided by the variant in which the dosing needle is placed at a minimum distance from the examined surface, but does not touch the drop lying on the surface of the sample.

#### 3.4.3. Minimizing Errors in Determining Contact Angles

As mentioned in the Introduction, semi-solid gel-like IEMs are difficult objects to measure contact angles. Their surface is nanoporous, geometrically and chemically heterogeneous, capable of restructuring upon contact with a liquid. The humidity of the IEM during measurements can be ensured by using a wet substrate connected by a wick to a vessel filled with water (Section 3.4.1, Figure 4b).

**Figure 7:**
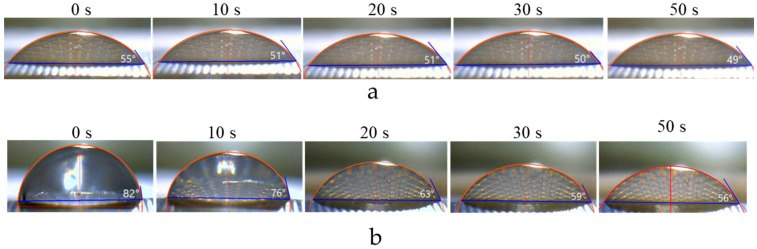
Transformation of the image of a sessile drop as a function of the time as it reached the surface of a wet (**a**) and dry (**b**) CJMC-3 sample.

**Figure 8:**
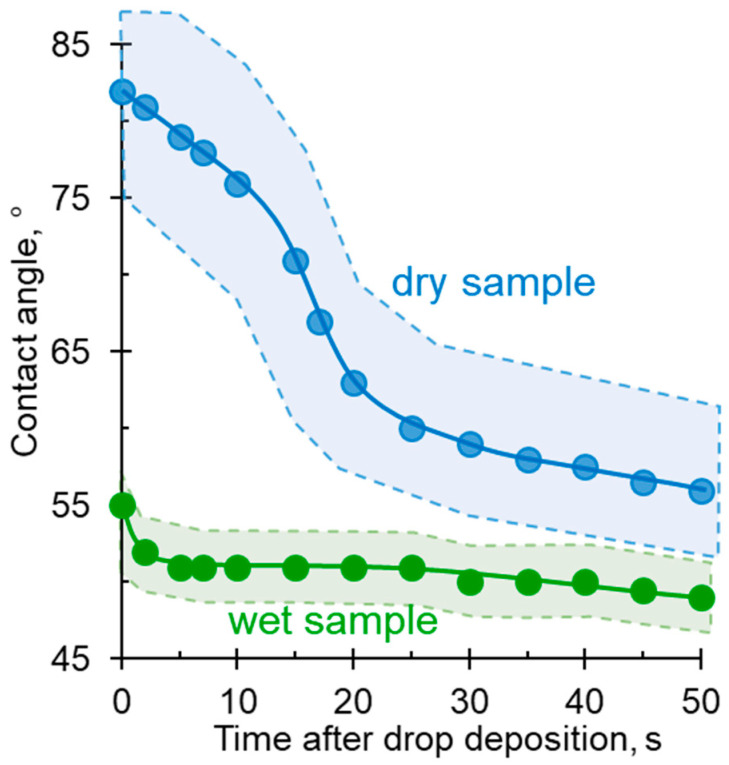
Contact angle values vs. time after sessile of a drop to the surface of a wet and dry CJMC-3 sample. The shaded area shows the confidence interval.

Figure 7 and Figure 8 show that, when a drop of water is applied to the surface of a wet membrane, the contact angle is already set after 5 s. The value of *θ_ap_* slightly decreases in time in the interval from 10 s to 50 s (51 to 49 deg); at the same time, the drop size noticeably decreases due to its drying. In the case of a drop falling on a dry membrane, the contact angle decreases much faster and the droplet size decreases considerably, because, in addition to its drying, water is also absorbed in the membrane.

Thus, the use of dry membranes does not allow obtaining adequate contact angle results. Keeping the IEM wet makes it possible to obtain reasonable values of *θ_ap_*, with the recommended time interval for measurements being from 5 to 15 s (Figure 8). An important prerequisite for such measurements is also the use of a pressure plate (Figure 4b), which prevents the membrane from deforming during measurement and improves the contact between the membrane and the wet substrate. According to our estimates, the use of the pressure plate reduces the relative measurement error from 15% to less than 5%.

## 4. Results and Discussion

### 4.1. Membranes with Identical Characteristics of Both Surfaces: Influence of Geometrical and Chemical Heterogeneities

**Figure 9:**
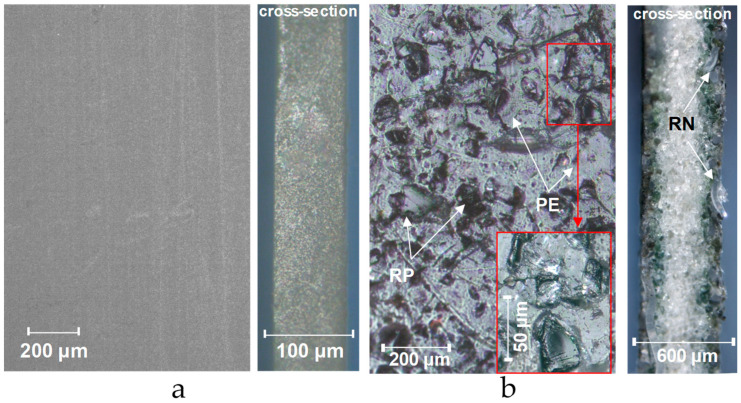
Optical images of the surface and cross-sections of an unreinforced homogeneous CJMA-4 membrane (**a**) and a heterogeneous MA-41 membrane (**b**). RP is an ion-exchange resin particle; RN is a filament of reinforcing net; PE is a polyethylene binder.

**Figure 10:**
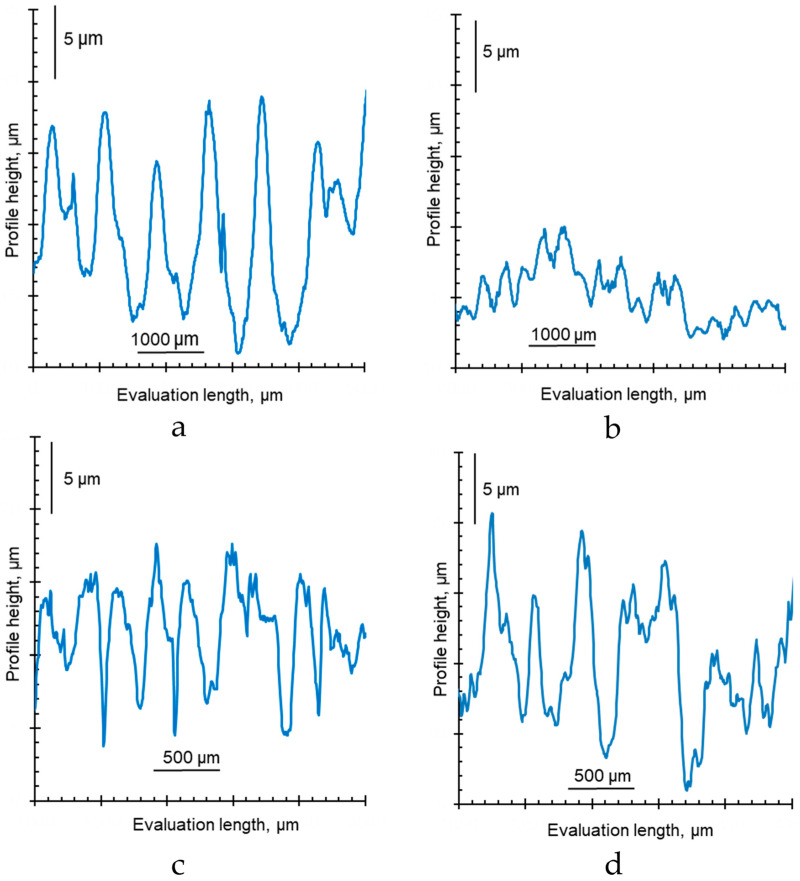
Surface profiles of swollen ion-exchange membranes CMX (**a**), AMX (**b**), MK-40 (**c**) and Ralex AMH PES (**d**).

The optical images of the surfaces and cross-sections of some IEMs are shown in Figure 9. Examples of surface profilograms are shown in Figure 10. The obtained values of contact angles and roughness parameters are summarized in Table 4.

CJMC-4, CJMA-4 and Neosepta CSE membranes contain no reinforcing material. Their surface topography is, most likely, mainly determined by defects in the substrate on which the copolymerization of the monomers takes place (casting method [64,66]). These membranes have the smallest deviations of the r value from unity.

Swollen homogeneous Neosepta CMX, AMX and ACM membranes have an undulating surface; the maximum distance between adjacent hills (parameter *S_m_*, Table 4) is reached for the AMX membrane and does not exceed 880 ± 240 µm. The profile height (*R_t_*) on average does not exceed 29.3 ± 2.5 μm. Similar parameters for the CMX and AMX membranes were obtained by Mareev et al. [69]. It is noteworthy that the distance between the deepest valley and the highest hill (*R_t_*) in the CMX is almost twice as big as in the AMX membrane (Figure 10a,b, Table 4), despite the fact that both membranes are made with the same paste method [84]. This method consists of applying a paste composed of monomers, polymerization initiators and polyvinyl chloride (PVC) powder to a PVC fabric for copolymerization. Sulfonic acid (CMX) or ammonium groups (AMX) are then introduced into the resulting membrane.

The surface of membranes made by the paste method is electrically homogeneous, and the size of polymer inert binder particles protruding on the surface does not exceed 100 nm, and this binder appears on the surface only after operation of such membranes in an electric field [85]. The difference in roughness parameters seems to be due to the fact that the exchange capacity (concentration of fixed groups) of CMX is 30% higher than that of AMX (Table 1). The hydration of these fixed groups provides a stronger swelling of the CMX ion-exchange material. The reinforcing fabric located near one of the membrane surfaces restrains the increase in linear dimensions of this (and other similar membranes) in length and width [69]. The increase in the volume of the ion-exchange material during swelling leads to the bending of the opposite surface, near which there is no reinforcing fabric. Since the exchange capacity of CMX is larger and, accordingly, the swelling of this membrane material is stronger, it has a more pronounced roughness than AMX in the swollen state. At the same time, in the dry state, both IEMs have the same roughness parameters [69].

As one would expect, the CMX membrane, which has a higher concentration of fixed groups (Table 1), shows a lower *θ_ap_* value compared to the AMX membrane (Table 4). The CJMA-4 membrane has the highest *θ_ap_* value (Table 4); this membrane is characterized by the lowest exchange capacity and it is made of a rather hydrophobic fluorine-containing material (Table 1).

Heterogeneous membranes MK-40, MA-41, Ralex AMH-PES (as well as Ralex CMH-PES, whose characteristics are given in Table 5) are made by hot rolling of milled ion-exchange resin and inert binder (polyethylene), and then both sides are pressed with a reinforcing fabric made of polymer threads [86]. The electrical heterogeneity of the surface of these membranes is mainly determined by the release of the ion-exchange resin granules protruding over the smoother polyethylene sections. In the case of MK-40 and MA-41, the average diameter of these granules, *d_av_*, ranges from 5 μm [87] to 19 μm [88]; the average distance between conductive surface areas, *l_av_*, varies from 9 μm [88,89] to 20 μm [62] and even 30 μm [87]; the protrusion of granules above the surface does not exceed 3 μm [62,89]. When fabricating Ralex AMH-PES and MA-41, as well as Ralex CMH-PES and MK-40, similar materials are used (Table 1), but in the case of Ralex membranes, the resin grind is more homogeneous: *d_av_* =5 μm [90]–10 μm [88]; *l_av_* =5 μm [88]. As a result, the fraction of the conductive surface that contains hydrated fixed groups ranges from 0.27 [88] to 0.60 [90] in the case of Ralex AMH PES, whereas for MA-41 this parameter ranges from 0.15 [90] to 0.23 [62]. A higher proportion of hydrophilic material predetermines the lower values of the contact angle of the Ralex AMH PES membrane compared to MA-41 (Table 4). The situation is similar when comparing Ralex CMH-PES and MK-40. 

The regularly repeated hills and valleys on the surface of all heterogeneous membranes recorded by profilometry have *S_m_* values (Table 4), which coincide with the distances between the intersections of reinforcing fabric filaments (Figure 10c,d). The *R_t_* value for heterogeneous membranes does not exceed 37.9 ± 7.1 µm. We (Figure 9b) and other researchers [91,92] sometimes observe threads coming to the surface in the places of intersections of reinforcing fabric filaments.

It is important to note that for all investigated membranes, the length of the contact line scanned with the profilometer does not exceed 1% compared to its projection (Parameter *r*, Table 4). Thus, the increase in the real surface profile compared to its projection is insignificant. The average length of elementary regions of all kinds of electrical and geometric heterogeneities on the surface of the swollen IEMs under study (*l_h_*) turns out to be 5–30 times lower than the diameter of the sessile drop base (*d_dr_* = 4000–7000 µm). Hence, these values satisfy the known requirements [52,93], according to which, in order to correctly measure the contact angle of inhomogeneous surfaces, the *d_dr_*/*l_h_* ratio must be > 3.

### 4.2. Membranes with Unequal Characteristics of Both Surfaces

**Figure 11:**
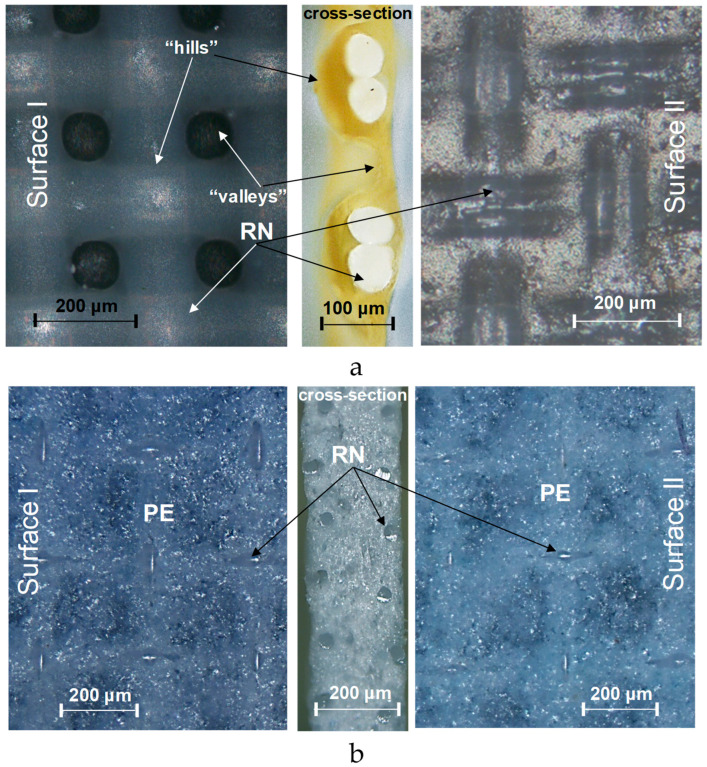
Optical images of surfaces I and II and cross section of homogeneous CJMA-3 membrane (**a**) and heterogeneous Ralex CMH membrane (**b**). RN is a filament of reinforcing fabric; PE is a polyethylene binder.

**Figure 12:**
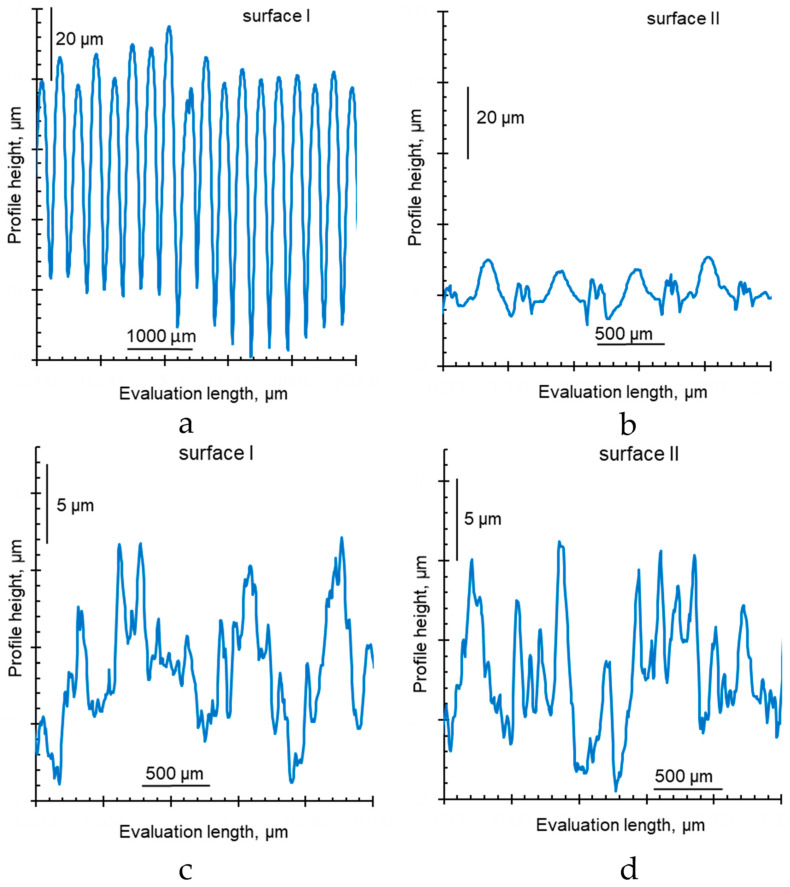
Profilograms of the surfaces of swollen ion-exchange membranes CJMA-3 (**a**,**b**) and Ralex CMH PES (**c**,**d**).

**Figure 13:**
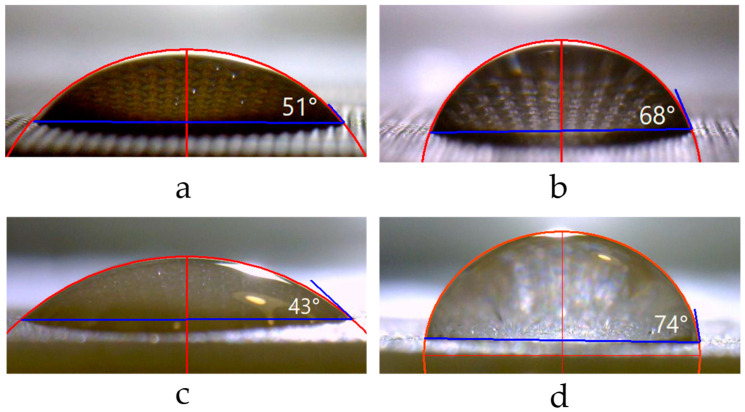
Images of droplets on the surfaces of the studied swollen membranes with unequal surfaces CJMA-3—I (**a**), CJMA-3—II (**b**), Ralex CMH PES—I (**c**) and Ralex CMH PES—II (**d**).

Figure 11, Figure 12 and Figure 13 show optical images of surfaces and cross sections, as well as profilograms and contact angles of some IEMs, the characteristics of one and the other surfaces of which are not identical. The roughness parameters and contact angles of such membranes are summarized in Table 5.

The reinforcing fabric of the CJMA-2, CJMA-3, CJMC-2 and CJMC-3 membranes is localized closer to one of their surfaces; let us designate this surface as “surface II”. Figure 11a shows the case of CJMA-3 membrane. On “surface II” of this membrane, the intersection of the reinforcing filaments is under a layer of ion-exchange material with a thickness of about 15 μm. The height of the profiles on this surface (*R_t_*) is 20.4 ± 3.2 µm. “Surface I” is more undulating, similar as in the case of CMX and AMX membranes considered above and in Ref. [69]. The profile height on this surface is 89.5 ± 3.9 µm.

The *S_m_* values, found from profilograms and presented in Table 5, are determined by the cell pitch of reinforcing fabric, equal on average to 280 ± 20 µm.

The difference in the maximum profile heights of the surfaces I and II of the studied membranes ranges from 4 (CJMC-2 and CJMC-3) to 70 μm (CJMA-3). The difference in roughness parameters of surfaces I and II increases with decreasing thickness and decreasing swelling ability of the ion-exchange material (Figure 12, Table 1).

It is important to emphasize that the values of the contact angles of the more embossed surface I of CJMA-2, CJMA-3, CJMC-2 and CJMA-3 membranes are smaller compared to the smoother surface II (Table 5). Since the surface of homogeneous IEMs can be assumed to be chemically homogeneous, the contribution to the difference in the contact angles of both surfaces may be due to geometric heterogeneity. Estimates of the Young’s contact angle (Table 5) made using Equation (2) allow us to conclude that any appreciable contribution of roughness, which was estimated as an increment in the contact line length to the value of contact angle, takes place only in the case of CJMA-3. Moreover, taking into account parameter r for this membrane leads to an increase in contact angle for the rougher surface II by only 3 degrees, while the difference between the contact angles of surfaces I and II reaches 17 degrees (Figure 13a,b, Table 5). Apparently, the main cause of the low contact angles on the surface I of ion-exchange membranes is the greater presence of water-filled mouths of micro- and macropores on the surface. The permselective micropores are enclosed within ion-exchange material, and the macropores are formed between the ion-exchange material and the threads of the reinforcing material, as was shown in [64]. The difference in the presence of charged pores on the IEM surfaces may be due to the peculiarities of manufacturing: during polymerization, one surface of the membrane faces the air, while the other faces the substrate. This can affect the orientation of the IEM functional groups on both surfaces, as it was reported by Ibanez et al. [94]: the air-facing side is enriched with fragments of hydrophobic sites, while the substrate-facing side is enriched with polar groups. However, we cannot know in advance which side of the available sample was facing the air and which was facing the substrate. Therefore, it is impossible to check this correlation in our case.

The Ralex CMH PES membrane shows close roughness parameters on both surfaces of the test sample. At the same time, the measured contact angles of surfaces I and II differ by 30 degrees (Figure 13c,d). These differences may be caused by the fact that (a) more reinforcement fabric “escapes” on surface I compared to surface II and (b) the fraction of hydrophobic inert binder on surface II is greater than on surface I. The occurrence of both these factors can be traced on the optical images of Ralex CMH PES surfaces (Figure 11b) and can be caused by differences in the conditions of its manufacturing process. Note that the characteristics of both surfaces of the Ralex AMH PES membrane are the same within the error of the experimental methods used.

As noted above, the contact angle of the surface can be used as a sensitive signal of the change in surface properties resulting from modification. Membrane modification is an effective way to improve the characteristics of commercial samples [23]. For example, surface modification of MK-40 membrane by LF-4SK layer with SiO_2_ nanoparticles embedded in it leads to an increase in the mass transfer rate due to the development of electroconvection [95]. This modification also causes a 5° decrease in the surface contact angle compared to the pristine membrane. The application of the modifier leads to smoothing of the surface topography (*R_t_* = 8.4 ± 3.0 μm, *R_a_* = 1.5 ± 0.2 μm, *S_m_* = 382 ± 44 μm) compared to the original characteristics (Table 4) A bulk modification of CJMA-3 membrane with polyquartenium-22, undertaken to reduce water splitting rate [96], does not cause significant changes in surface topography, but leads to a significant decrease in contact angles. For example, for surface I, the contact angle of the modified membrane was 35 ± 5°, which is lower than the value for the pristine membrane by 16° (Table 5).

## 5. Conclusions

This study proposed a new equipment and optimized protocol for measuring the contact angles of wet ion-exchange membranes using a simple sessile drop method. This allowed obtaining reliable and reproducible data under conditions when the membrane surface does not dry out or deform. The optimized size of the needle and the rate of water dosing ensured the reproducible water drop formation. The diameter of the drop was significantly greater than the size of heterogeneities on the surface, so that the contact angle does not depend on the drop localization on the membrane surface. Since there is currently no unified protocol for measuring contact angles of the IEM, this protocol can serve as the basis for developing a standard measurement procedure.

The validation of the proposed protocol for a large number of commercial ion-exchange membranes was presented. The results of surface characterization of the studied IEMs using optical microscopy and profilometry were provided as well. It was found that the contribution of the surface roughness of the studied membranes to the value of the contact angle was insignificant. For the CJMA-3 membrane, the roughest of the studied membranes, this contribution led to an increase in θ by only 3 degrees. A general correlation between the contact angle and the membrane exchange capacity (volume concentration of fixed charged groups) was established: the greater the exchange capacity, the smaller the contact angle. However, the contact angle depends also on the conditions of the membrane synthesis. For example, for CJMA and CJMC membranes, the contact angle for one side of the membrane and its other side was different, because one side was exposed to air during the drying of the membrane after preparation, while the other was in contact with a solid substrate. For a Ralex CMH PES membrane, different contact angles for one and the other side were explained by the conditions of hot pressing, in which more polyethylene binder appeared on one side of the membrane than on the other. Such information can be very useful in analyzing the reasons for the difference in the behavior of the membranes when they are oriented differently in the membrane stack.

## Figures and Tables

**Table 1:** Characteristics of the membranes under study.

Manufacturer	Membranes	Ion Exchange Matrix	Functional Groups	Inert Binder	Reinforcing Material	Thickness of Wet Membrane, Microns	Water Content, gН_2_О/g Dry, %	Exchange Capacity, mmol g^−1^ Wet
Homogeneous membranes								
Astom, Japan	CMX	DVB + PS	$-SO_{3}^{-}$	PVC	PVC	175 ± 2	23.2 [60]	1.61 ± 0.05 [60]
	CSE	no data		no data	-	141 ± 2	42 [61]	1.85 [61]
	AMX	DVB + PS	$-N^{+}{\left(CH_{3}\right)}_{3}$	PVC	PVC	127 ± 1	14.4 [60]	1.23 ± 0.05 [60]
	ACM	no data	no data	no data	no data	101 ± 1	13.9 [62]	1.58 [62]
Hefei Chemjoy Polymer Material, China	CJMA-2	no data	$-N^{+}{\left(CH_{3}\right)}_{3}$	no data	no data	141 ± 2	35 [63]	0.8–1.0 [63]
	CJMA-3	PVDF		no data	PET	143 ± 2	17 [64]	0.57 ± 0.05 [64]
	CJMA-4			no data	-	98 ± 1	15–20 [65]	0.5–0.6 [65]
	CJMC-2		$-SO_{3}^{-}$	no data	no data	161 ± 2	200 [63]	0.8–1.0 [63]
	CJMC-3			no data	polyester	183 ± 2	44 ± 5 [66]	0.63 ± 0.05 [66]
	CJMC-4			no data	-	118 ± 1	35–40 [65]	0.8–1.0 [65]
Heterogeneous membranes								
Shchekinoazot, Russia	MK-40	DVB+PS	$-SO_{3}^{-}$	PE	nylon	525 ± 3	25.7 [60]	1.43 ± 0.08 [60]
	MA-41		$-N^{+}{\left(CH_{3}\right)}_{3}$			485 ± 3	19.1 [60]	1.22 ± 0.06 [60]
MEGA, Czech Republic	CMH PES		$-SO_{3}^{-}$		polyester	557 ± 2	31 [67]	2.34 [67]
	AMH PES		$-N^{+}{\left(CH_{3}\right)}_{3}$			585 ± 2	56 [67]	1.97 [67]

DVB + PS is copolymer of polystyrene and divinylbenzene; PVC is polyvinyl chloride; PE is low-pressure polyethylene; PVDF is polyvinylidene fluoride; PET is polyethylene terephthalate.

**Table 2:** Main factors influencing the result of contact angle measurement using the sessile drop method and optimized experimental conditions.

Factors and Parameters		Optimized Equipment, Conditions and Parameter Values
Configuration and parameters of the measuring system	Sample preparation	Salt pretreatment procedure and equilibration with working solution
	Liquid dosing system	A syringe pump connected to a dispensing needle (needle tip shape pst3, size 20G). Fluid dispensing rate W = 55 µL/min (corresponds to a droplet volume of 14.4 µL)
	Tilt angle of the microscope lens	1.5°
	Background lighting	LED source with a diffuser located behind the sample
	Distance between needle and sample	4–5 mm
Protocol for image registration	Number of measurements	>20 on different sample areas. Processing by Student’s t-test using Grubbs criterion
	Measurement time (time to reach steady state)	From 5 to 15 s
Contact angle acquisition	Image processing method	Fitting method (RisingView software) Contact angles obtained from both sides of the drop are averaged

**Table 3:** Experimental (*θ_ap_*) and reference (*Ɵ_A_,* *Ɵ_R_*) * values of the contact angles of the surface of standard materials.

Material	Distance between Needle and Sample, mm	*R_a_*, μm	Experimental Contact Angle *θ_ap_*, Degree	Reference Contact Angle, Degree
Polytetra-fluoroethylene	4	0.4	108.5 ± 0.2	*Ɵ_A_* = 108.9 *Ɵ_R_* = 96.0 [83]
	5		108.3 ± 0.5	
	6		108.3 ± 0.6	
Polymethyl-methacrylate	4	0.03	74.6 ± 0.4	*Ɵ_A_* = 74.7 *Ɵ_R_* = 54.2 [83]
	5		74.9 ± 0.4	
	6		75.0 ± 0.5	
Paraffin	4	-	110.7 ± 0.2	*Ɵ_A_* = 116 ± 2 *Ɵ_R_* = 92 ± 3 [40]
	5		110.8 ± 0.2	
	6		109.6 ± 0.6	

* *Ɵ_A_* and *Ɵ_R_* are the advancing and receding contact angles, respectively.

**Table 4:** Contact angles and roughness characteristics of swollen IEM with identical characteristics of both surfaces.

Membranes	*θ_ap_*, °	*r*	*R_t_*, μm	*R_a_*, μm	*S_m_*, μm
Neosepta CMX	61 ± 1	1.0020 ± 0.0005	29.3 ± 2.5	3.7 ± 0.3	640 ± 50
Neosepta CSE	53 ± 2	1.0001 ± 0.0001	5.0 ± 1.1	0.5 ± 0.3	500–1000
Neosepta AMX	68 ± 1	1.0003 ± 0.0001	9.7 ± 1.7	1.9 ± 0.3	880 ± 240
Neosepta ACM	66 ± 1	1.0005 ± 0.0001	12.2 ± 1.7	1.9 ± 0.2	548 ± 74
CJMC-4	64 ± 1	1.0001 ± 0.0001	4.5 ± 2	0.9 ± 0.2	500–1000
CJMA-4	78 ± 1	1.0001 ± 0.0001	4.7 ± 1.2	0.8 ± 0.2	500–1000
МК-40	70 ± 1	1.0032 ± 0.0005	13.5 ± 4.3	1.9 ± 0.1	245 ± 11
МА-41	69 ± 1	1.0027 ± 0.0004	15.5 ± 3.5	1.3 ± 0.1	226 ± 18
Ralex AMH PES	53 ± 3	1.0068 ± 0.0009	36.5 ± 6.3	4 ± 0.5	357 ± 58

**Table 5:** Contact angles and geometric heterogeneity characteristics of IEM with different surfaces.

Membranes	Surface	*θ_ap_*, °	*θ^Y^*, °	*r*	*R_t_*, μm	*R_a_*, μm	*S_m_*, μm
CJMA-2	I	72 ± 2	72	1.017 ± 0.005	48.0 ± 3.0	8.5 ± 1.2	280 ± 15
	II	74 ± 1	74	1.0002 ± 0.0002	11.6 ± 4.1	1.8 ± 0.3	600 ± 50
CJMA-3	I	51 ± 2	54	1.066 ± 0.006	89.5 ± 3.9	15.1 ± 0.5	284 ± 6
	II	68 ± 2	68	1.007 ± 0.002	20.4 ± 3.2	2.5 ± 0.4	251 ± 48
CJMC-2	I	67 ± 1	67	1.0006 ± 0.0002	13.3 ± 7.2	2.1 ± 0.7	306 ± 41
	II	73 ± 2	73	1.0003 ± 0.0002	8.9 ± 2.4	1.1 ± 0.2	303 ± 70
CJMC-3	I	51 ± 2	52	1.006 ± 0.004	19.2 ± 2.6	2.9 ± 0.4	280 ± 20
	II	60 ± 1	60	1.0016 ± 0.0002	15.4 ± 4.8	2.8 ± 0.4	285 ± 27
Ralex CMH PES	I	43 ± 5	44	1.0079 ± 0.0011	16.7 ± 4.2	3.6 ± 0.4	303 ± 35
	II	74 ± 5	74	1.0076 ± 0.0008	21.6 ± 3.4	3.4 ± 0.5	296 ± 92

*θ^Y^* is Young’s contact angle determined according to Equation (2).
